# CBCT Evaluation and Conservative Management of a Large Periapical Lesion Associated with Dens Invaginatus Type II

**DOI:** 10.1155/2022/1529835

**Published:** 2022-02-14

**Authors:** Laïla Azzahim, Nisrin Bassim, Faïza Abdallaoui

**Affiliations:** Faculty of Dentistry of Rabat, Mohamed V University, Rabat, Morocco

## Abstract

This case report illustrated the conservative management of an invaginated type II tooth with a large periapical lesion. This dental malformation is characterized by the complexity of root canal anatomy, which when associated with a periapical lesion complicates the performance of conventional endodontic treatment, hence resulting to the difficulty of obtaining the optimal disinfection of the root canal system, which is an essential element for the success of any endodontic treatment. In the present case report, clinical and radiographic examinations were supplemented by CBCT examination to identify the root canal configuration and the extension of the periapical lesion. Conservative orthograde endodontic treatment was performed by combining mechanical and chemical action. Additionally, active nonsurgical decompression was performed and the follow-up visits revealed a favorable outcome.

## 1. Introduction

Dens invaginatus (DI), also known as dens in dente, is a dental malformation found mostly in the permanent dentition and has been reported to affect 0.3% to 10% of the population [1]. According to the most widely accepted theory, it results in an invagination of the enamel organ into the dental papilla prior to the calcification of dental tissues [2]. The maxillary lateral incisor is the most commonly affected tooth [3]. The root canal system is characterized by anatomic complexity. Thus, the diagnosis and treatment planning are considered difficult [4].

This case report showed the importance of the use of Cone Beam Computer Tomography (CBCT) evaluation to facilitate the exploration and accessibility of the complicated root canal system of the invaginated tooth. In addition, it demonstrated that the use of the orthograde conservative approach alone allowed the healing of the periapical lesion despite the complexity of the root canal anatomy and the wide extension of the periapical lesion.

## 2. Case Presentation

A 34-year-old male patient, with good general health, was referred for endodontic treatment of the maxillary lateral right incisor (tooth #12). The patient had priorly received an endodontic treatment by a general practitioner. The patient had been complaining of pain associated with recurrent palatal swelling on the right side of the palate for 6 months. There was no history of trauma.

**Figure 1:**
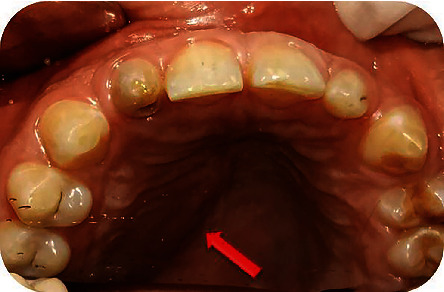
Preoperative photograph showing a palatal swelling on the right side.

**Figure 2:**
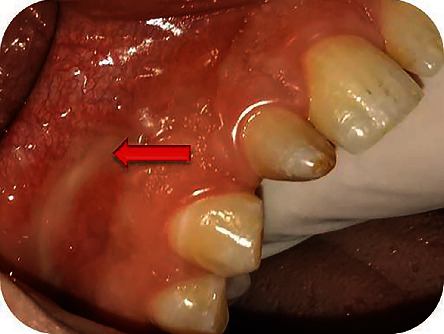
Drainage of pus from the vestibular fistula proximal to the apical area of tooth #12.

The extraoral examination revealed no abnormalities. The intraoral examination showed tooth #12 as a “rice-shaped tooth,” a palatal swelling on the right side (Figure 1), and a fistula in the alveolar mucosa, proximally to the apical area of tooth #12. Response to pulp sensitivity test was negative, while both vertical and horizontal percussions were positive. There was no increased tooth mobility or probing depth. Palpation at the level of palatal swelling was painful and allowed drainage of pus from the vestibular fistula (Figure 2).

**Figure 3:**
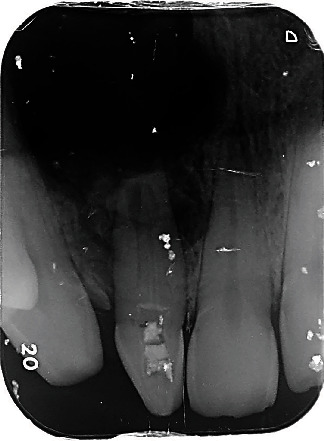
Preoperative radiograph showing a complex root canal configuration corresponding to that of type II DI associated with a large extending periapical image in relation to tooth #12.

**Figure 4:**
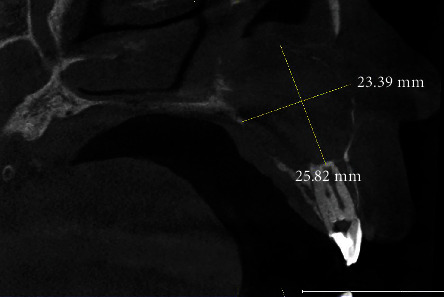
CBCT sagittal section confirmed that it is a type II DI, showing a wide radiolucent periapical image measuring 25.82 mm × 23.39 mm communicating with the nasal cavity.

**Figure 5:**
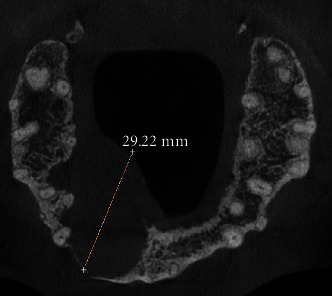
CBCT axial section showing the rupture of the internal and external bony cortices caused by the extension of the periapical lesion.

The radiographic examination showed the presence of DI; the invagination extended from the crown to the root apex, associated with a radiolucent lesion in the periapical area of tooth #12 (Figure 3). Cone Beam Computer Tomography (CBCT) scan showed the presence of type II invagination communicating with the root canal and revealed a large periapical radiolucency related to tooth #12 (Figures 4 and 5). The diagnosis was chronical apical periodontitis related to tooth #12 with an infected DI type II. The treatment plan was to perform an orthograde endodontic treatment.

After obtaining the patient's informed consent, a rubber dam was applied, the access cavity was modified, and the root canal and the invagination were located. The working length was measured using an apex locator and confirmed with an X-ray examination. Both the canal and the invagination were instrumented through a crown-down technique using manual instruments. In order to overcome the lack of mechanical instrumentation in the presence of complex root canal anatomy, all attention has been paid to chemical disinfection. Irrigation used was 2.5% Sodium Hypochlorite (NaOCl) for a total time of 30 minutes for the root canal and the invagination. NaOCl was conveyed into the canal using an irrigation syringe with a side outlet needle, the solution was renewed regularly. To improve disinfection, sonic activation was used with the EndoActivator sonic device (Dentsply Sirona) for 30 seconds. NaOCl irrigation was alternated with 17% Ethylene Diamine Tetra acetic Acid (EDTA) gel to dissolve inorganic substances. The active nonsurgical decompression with an irrigation syringe was performed with the objective of aspirating as much as possible the intracanal serosities. Then, the intracanal dressing with calcium hydroxide (Ca(OH)_2_) paste was placed for 2 weeks. The access cavity was sealed with a temporary restorative material. Two weeks later, the tooth was asymptomatic. Since the intracanal serosities did not dry up, the intracanal medication was renewed. After a month, the tooth became asymptomatic and the canal dry. Then, root canal filling was performed by a lateral condensation technique associated with thermomechanical compaction using gutta-percha cones and the access cavity was restored using a laminate technique (glass ionomer cement-light-cured composite resin).

**Figure 6:**
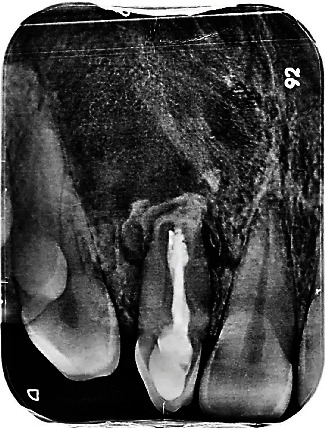
18-month follow-up radiograph showing signs of bone neoformation.

Clinical and radiographic control sessions were carried out at 6, 12, and 18 months after the treatment. During the follow-up period, no signs and symptoms related to the concerned tooth were reported by the patient and bone neoformation was noticed in the periapical area (Figure 6).

## 3. Discussion

The endodontic management of DI is a challenge due to the complexity of the root canal anatomy. Several classifications of DI have been proposed; the most commonly used one was proposed by Oehlers [5]. According to this classification, there are 3 types of invagination [5, 6].

- Type I shows an invagination limited to the crown
- Type II extends below the cementoenamel junction, ends as a blind sac, and may or may not have communication with the pulp
- Type III extends through the root and perforates the apical (type IIIa) or lateral area (type IIIb), without any immediate communication with the dental pulp [5]

When this complicated canal configuration (type II or III) is associated with an extensive periapical lesion, nonsurgical endodontic treatment is difficult to perform, but it must be considered before any surgical treatment [3]. The use of CBCT as an auxiliary tool is recommended for the diagnosis and the treatment planning of teeth with such developmental anomalies [7].

Root canal can be instrumented with manual or rotary files while it is recommended to shape the invagination with manual instruments to prevent the occurrence of instrumental fractures with rotary files within the invagination since the surface is predominantly covered by enamel and has varying shapes [4]. The use of XP-endo Finisher was also reported to shape the root canal in a maxillary lateral incisor presenting type II [8]. In the present case, both the root canal and the invagination were shaped with hand NiTi endodontic instruments according to the crown-down concept. It is important to increase the chemical disinfection alternating abundant 2.5% NaOCl irrigation and EDTA gel to compensate for the insufficient mechanical instrumentation due to the complex canal anatomy [9]. NaOCl has bactericidal, solvent effect for organic material and lubricating action [10]. The use of ultrasound is essential in optimizing disinfection [8] and can also be supplemented with Ca(OH)_2_ medication [11]. Although this medication is currently controversial, its use in this case was justified by the presence of abundant serosities and the need to alkalize the inflammatory medium created by the periapical reaction of the deep periodontium, in addition to its antibacterial, hemostatic, and osteogenic properties [11]. Another molecule that can be used in intracanal medication is the 2% chlorhexidine gel. It has a bactericidal action on bacteria resistant to Ca(OH)_2_ by remanence effect for at least two weeks, but its parietal elimination is difficult [12].

The success of endodontic therapy can be judged clinically by the absence of symptoms (pain, infection, and fistula) and radiographically by the decrease in bone lesion size, osteoformation, and reconstitution of the lamina dura [13]. The presented case was followed up clinically and radiographically for 18 months, indicating the absence of clinical symptoms associated with regression of the radiolucent image size, which was considered a success of endodontic orthograde therapy. Indeed, studies have shown that 90% of periapical lesions show signs of healing after 1 year when the majority of lesions heal at 2 years, but in some cases, the disappearance of the lesion can take 4 to 5 years. Generally, the success of endodontic treatment is judged during the first year of follow-up [13].

### 3.1. The Clinical Relevance

The importance of combining mechanical action with chemical disinfection has been widely proven to eradicate endodontic infection and can be effective without resorting to endodontic surgery which is mutilating to periapical tissue even with complex anatomy.

## 4. Conclusion

In the light of the findings of the present case report, three points need to be stressed:

- The presence of a complex root canal anatomy does not systematically justify the use of apical surgery
- Orthograde endodontic treatment is the first-line treatment for the management of large extended periapical lesions. Only if this fails, other options should be considered
- The mechanical instrumental action is limited by the presence of a complex root canal anatomy. To compensate for this limitation of mechanical action, contact time of the chemical solution should be extended and more molecules should be added

## Data Availability

The data used to support this case report are available from the corresponding author on reasonable request.
